# Relationship Between Social Determinants of Health and Domains of Care Addressed During Pediatric Palliative Care Visits for Children with Cancer

**DOI:** 10.3390/children12121694

**Published:** 2025-12-16

**Authors:** Deborah Feifer, Hee Su Park, Katherine Lee, Linda Radbill, Khaliah Johnson, Dio Kavalieratos, Katharine Brock

**Affiliations:** 1 Doctor of Medicine Program, Emory University School of Medicine, Atlanta, GA 30322, USA; 2 Aflac Cancer and Blood Disorders Center, Children’s Healthcare of Atlanta, Atlanta, GA 30329, USA; 3 Department of Pediatrics, Emory University School of Medicine, Atlanta, GA 30322, USA; 4 Division of Palliative Care, Children’s Healthcare of Atlanta, Atlanta, GA 30329, USA; 5 Department of Family and Preventative Medicine, Emory University School of Medicine, Atlanta, GA 30322, USA

**Keywords:** pediatric palliative care, social determinants of health, health disparities, pediatric oncology

## Abstract

**Highlights:**

**What are the main** **findings?**

• Although disparities exist in pediatric palliative care (PPC) by social determinants of health, domains of care addressed in PPC visits were similar across patient race/ethnicity, social deprivation index score, primary language, and patient/clinician race concordance.
• Some differences in subdomains emerged, with symptoms (e.g. nausea/vomiting) more likely to be addressed in Non-Hispanic White visits than other races/ethnicities.

**What are the implication of the main findings?**

• Disparities in PPC and end-of-life (EOL) outcomes likely emerge from a complex interplay of variables beyond visit content, including health literacy, patient preference, historical context, and systemic factors.
• Clinicians can attempt to mitigate disparities by ensuring proactive symptom management and goal concordant care, and researchers can further investigate how PPC visit content influences patient outcomes.

**Abstract:**

**Introduction:** Pediatric palliative care (PPC) improves symptom management and end-of-life (EOL) outcomes. Disparities exist in access to PPC and EOL care related to social determinants of health. Less is known regarding how the content of PPC visits varies by sociodemographic factors like race/ethnicity, socioeconomic status, and language. **Methods:** This retrospective cohort study included patients 0–27 years old with cancer receiving PPC between 2017 and 2022. After each PPC visit, the documenting clinician selected the domains of care addressed during the visit (Goals of Care, Symptom Management, and Care Coordination with respective subdomains). Differences in frequency of subdomains discussed were compared across patient race/ethnicity, social deprivation index (SDI) score, language, and concordance with clinician race/ethnicity. Chi-square or Fisher’s exact test assessed differences in proportions of visits with each subdomain discussed, and Kruskal–Wallis tests assessed differences in the frequency of total subdomains discussed. **Results:** Among 467 patients, there were 7548 PPC visits. Most patients were non-Hispanic (n = 384, 82.2%), English-speaking (n = 425, 91.0%), and identified as White (n = 270, 57.8%) or Black (n = 166, 35.5%). A median of 8 (IQR 7, 11) subdomains were addressed per visit. One more subdomain was addressed in non-Hispanic White visits (9) compared to all other races/ethnicities (8, *p* < 0.001). Certain topics, like symptoms (e.g., nausea/vomiting), were more likely to be addressed in visits with White and Hispanic/Latino patients. One more subdomain was addressed in the intermediate disadvantage group (9, IQR 7, 11) compared to high and low disadvantage (8, IQR 7, 11) (*p* = 0.092). Both English- and non-English-speaking visits addressed a median of 8 subdomains (*p* < 0.001). One more subdomain was addressed in patient/clinician race-discordant (9, IQR 7, 11) than race-concordant encounters (8, IQR 7, 10) (*p* < 0.001). **Conclusions:** While EOL outcomes often differ for groups of different races, ethnicities, social deprivation indices, and languages, the frequency of subdomains discussed during PPC visits was fairly similar across groups. Disparities in PPC and EOL outcomes likely emerge from a complex interplay of variables beyond visit content, including health literacy, patient preference, historical context, and systemic factors.

## 1. Introduction

Children with cancer and their families navigate complex treatment decisions, symptom burden, and emotional distress throughout their cancer experience. Pediatric palliative care (PPC) aims to support patients and their families coping with serious illness, relieve suffering, and improve quality of life [1,2]. Patients who receive PPC are more likely to have improved symptom burden and pain control [3] and less likely to receive intensive treatment at the end of life (EOL) [4,5,6]. During PPC visits, clinicians address numerous domains of care, including Goals of Care, Advance Care Planning (ACP), Symptom Management, and Care Coordination [7,8]. The domains addressed during visits vary by patient needs, visit type [9], and proximity to EOL [8].

Despite the growth and value of PPC within pediatric oncology, disparities exist in patients’ ability to access and receive PPC and in EOL outcomes [10,11]. Among PPC recipients, these disparities have been attributed to non-modifiable, patient-level factors including race [12,13,14,15,16], ethnicity [17,18], language [17,18], and socioeconomic status (SES) [14,17,19,20]. White patients are more likely to receive palliative care than other races (Williamson), and Hispanic patients are less likely to receive inpatient PPC than non-Hispanic patients [17,18]. Parents with limited English proficiency are more likely to have a documented financial burden and limited health insurance coverage [21,22], and their children are more likely to undergo longer hospitalizations [21]. White patients are more likely to die at home [13,14] and spend fewer days in the hospital in the last 90 days of life [12]. Meanwhile, Black patients are significantly more likely to receive CPR [16] and to undergo more intense interventions at EOL [23]. Patients at lower SES are more likely to receive higher intensity care at EOL, including hospital death [14,19], and are less likely to receive PPC [17,20].

Preferences for care [19], psychological distress [24], prognostic awareness [25], and decision-making roles [26,27] also vary by sociodemographic factors, highlighting the complex and interconnected web of variables that affect outcomes in pediatric oncology [11]. While existing research emphasizes differences in receipt of PPC, EOL outcomes, and healthcare utilization [6,16], no studies have quantitatively examined whether the content of PPC visits for children with cancer differs across sociodemographic variables.

In this study, we assessed differences in the domains of care addressed during inpatient and outpatient PPC visits of children, adolescents, and young adults with cancer by social determinants of health, including race/ethnicity, primary language, and socioeconomic disadvantage. We also performed an exploratory analysis of whether racial concordance between the child with cancer and the PPC clinician affected the frequency of domains addressed during PPC visits. Understanding the content of PPC visits may enrich our understanding of existing disparities and help narrow these gaps in care.

## 2. Materials and Methods

### 2.1. Domains of PPC Addressed During Visits

At Children’s Healthcare of Atlanta, patients with cancer can be referred to either inpatient or outpatient PPC by their oncology, bone marrow transplant, or intensive care team. There are no specific consultation triggers. After each PPC visit, the inpatient or outpatient documenting physician or nurse practitioner (NP) selects the domains and subdomains addressed during the visit within the electronic health record (EHR) using a drop-down (select all that apply) menu. There are three main domains of care with associated subdomains: Goals of Care, Symptom Management, and Care Coordination. Goals of Care subdomains encompass a patient’s understanding of their illness and discussion regarding their preferences for care, including prognostic understanding, decision-making, factors of importance (e.g., spirituality), as well as discussion around ACP, code status, and use of technology as their disease progresses. Symptom Management subdomains address patient concerns regarding their physical (pain, nausea, fatigue) and emotional symptoms (anxiety, depression/sadness). Care Coordination subdomains involve collaboration between the palliative care team and other medical services (specialists, primary care physician, psychosocial care), hospice coordination, and community services (school issues, home care). The full subdomain list is available in the table in Section 3.2. A write-in option is also available within each section. K.E.B. recategorized write-in options into existing subdomains, or if a topic was new and recurring, added a new subdomain. Full methods have been previously published [8].

### 2.2. Retrospective Review

This study is a retrospective review of all billable PPC encounters that occurred between June 2017 and December 2022 (CHOA IRB 00001560). Eligible patients had an oncologic diagnosis, were treated at the Aflac Cancer & Blood Disorders Center during the study period, received inpatient and/or outpatient PPC, and were between the ages of 0 and 27. Visits without an oncologic diagnosis and/or where no bill was generated (e.g., acknowledgements of consult requests or visits by a social worker alone) were excluded. Demographic information, including age at diagnosis, sex, self-reported race, ethnicity, primary language, zip code at diagnosis, and vital status, was extracted from the EHR and the PPC clinic database.

Social disadvantage was measured with the social deprivation index (SDI), which is a validated composite measure that includes poverty level, employment, transportation, education level, housing, crowding, and family structure [28,29,30,31]. Patients were linked to their SDI score (ranging from 0 to 100) using their zip codes at diagnosis converted to zip code tabulation areas (ZCTAs). Patients were ordered from lowest to highest SDI and divided into tertiles (low, middle, and high disadvantage) based on the 33rd and 67th percentiles of the sample. SDI is more strongly associated with poor healthcare access and health outcomes than a measure of poverty alone (i.e., living below the poverty line) [29]. ZCTAs were also linked to their rural–urban commuting area (RUCA) codes, which use measures of population density, urbanization, and daily commuting to categorize U.S. census tracts [32]. Patients were classified as living in an urban or suburban/rural area at diagnosis [33,34].

Visits were further dichotomized and analyzed by race concordance between patient and clinician. PPC clinicians’ race was self-reported in a survey of all physicians, NPs, licensed clinical social workers (LCSWs), and fellows who conducted PPC visits between 2017 and 2022. During that time, the inpatient PPC team consisted of eight physicians (two non-Hispanic Asian, two non-Hispanic Black (NHB), and four non-Hispanic White (NHW)), five NHW NPs, and two NHW LCSWs. The outpatient PPC team had one NHW physician and one NHW NP. Patient race/ethnicity was identified using the National Childhood Cancer Registry, which collects self-reported demographic data from the EHR. Visits were documented as race-concordant if patients and clinicians were of the same race/ethnicity (i.e., an NHW clinician with an NHW patient) and were otherwise race-discordant (i.e., an NHW clinician with an NHB patient or an NHB clinician with an Asian patient). If multiple providers were present at the visit, the visit was documented as race-concordant if at least one provider was of the same race/ethnicity as the patient.

### 2.3. Statistical Analysis

Descriptive statistics were computed for all study variables, including frequencies for categorical variables and medians (interquartile range (IQR)) for continuous variables where appropriate. Within each domain, the number of subdomains discussed was summed by visit. Differences in the frequency and total number of subdomains discussed were compared across patient race/ethnicity, SDI, language, and concordance with clinician race/ethnicity. Pearson’s chi-square or Fisher’s exact test was used to assess differences in proportions of visits with the subdomain discussed, and Kruskal–Wallis tests were used to assess differences in the frequency of total subdomains discussed. All analyses were conducted using RStudio (v4.4.0) and tidyverse (v2.0.0), gtsummary (v2.0.3), and nlme (v3.1-164) packages [35,36,37,38]. *p*-values were two-sided. Results were considered statistically significant if *p* < 0.05.

Due to the high number of visits, some analyses may be overpowered and find statistical differences between groups even when the magnitude of the difference is negligible. Therefore, we established a threshold for clinical significance. Results were considered clinically significant if there was a ≥10% difference between groups [39].

## 3. Results

### 3.1. Patient Demographics

Of the 467 included patients, most were non-Hispanic (n = 384, 82.2%), English-speaking (n = 425, 91.0%), and living in urban areas (n = 419, 89.7%; Table 1). A majority of patients identified as White (n = 270, 57.8%) or Black (n = 166, 35.5%). Patients were a median of 10.4 years old at diagnosis. Diagnoses included central nervous system (CNS) tumor (n = 166, 35.5%), solid tumor (n = 170, 36.4%), and leukemia/lymphoma (n = 131, 28.1%). Based on assigned tertiles, approximately 33% of patients were in each SDI category (low, middle, and high disadvantage). Average SDI scores varied by patient race/ethnicity, with the highest scores (indicating greater disadvantage) in NHB (62.23) and Hispanic/Latino patients (53.33) compared to NHW (44.41) and Asian patients (38.89) (Supplemental Table S1).

### 3.2. Differences in Domains of Care by Race/Ethnicity

There were 7548 total visits with PPC, with a median of 10 visits per patient. Analyses compared domains of care addressed in visits with NHW patients (n = 3238), NHB patients (n = 2439), Hispanic/Latino patients (n = 1445), and patients identifying as Asian/Other (n = 426). The median number of subdomains discussed per visit was slightly higher for NHW visits (9) compared to all other races/ethnicities (8, *p* < 0.001; Table 2). There were statistically significant differences by race/ethnicity across many subdomains; however, fewer subdomains met the threshold for clinical significance, and these are denoted in each supplemental table.

**Figure 1:**
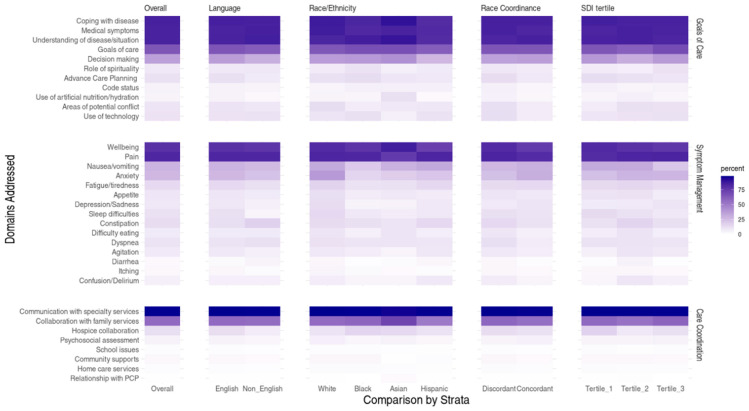
Comparison of PPC subdomains addressed by sociodemographic variables.

While the median Goals of Care subdomains discussed per visit were 4 (IQR 3, 5) for each race/ethnicity category, the decision-making subdomain was addressed in fewer visits with Hispanic/Latino patients (27% vs. 37–42%; *p* < 0.001). Symptom Management subdomains were addressed less often in visits with NHB (2, IQR 2, 4) and Asian patients (2, IQR 2, 3) compared to NHW (3, IQR 2, 5) and Hispanic/Latino patients (3, IQR 2, 4) (*p* < 0.001). Nausea/vomiting was less commonly addressed in visits with NHB patients (20% vs. 30–33%; *p* < 0.001). Psychological subdomains, like anxiety and depression/sadness, were addressed more often in NHW visits compared to other race/ethnicities (38% vs. 16–22% and 13% vs. 4.6–8.6%, respectively, *p*-values < 0.001). The frequency of Care Coordination subdomains was similar across all races/ethnicities (Table 2; Figure 1).

### 3.3. Differences in Domains of Care by Social Deprivation Index

Among all 7548 visits, there were 2318 (31%) visits with low-disadvantage patients, 2476 (33%) with middle-disadvantage, and 2754 (36%) with high-disadvantage patients. Across tertiles, there were no significant differences in the total subdomains addressed per visit (*p* = 0.092; Table 3). Tertiles 1 (low disadvantage) and 3 (high disadvantage) addressed a median of 8 subdomains (IQR 7, 11), and tertile 2 addressed a median of 9 (IQR 7, 11).

While not clinically significant, the role of spirituality (11% vs. 6.9–7.8%) was more often discussed in high-disadvantage visits, whereas ACP (13% vs. 9.9–11%) and code status (6.2% vs. 4.4–5.0%) were addressed slightly more often in low-disadvantage visits (*p*-values < 0.017). Nausea/vomiting, the only subdomain to reach our clinical significance threshold, was addressed less often in high-disadvantage visits (21% vs. 32%, *p* < 0.001). Hospice collaboration was addressed more often in low-disadvantage visits (17% vs. 8.7–12%, *p* < 0.001) (Figure 1; Supplemental Table S2).

### 3.4. Differences in Domains of Care by Language

In total, there were 6700 (89%) visits with English-speaking families and 799 (11%) visits with non-English-speaking families. Both groups addressed eight subdomains during visits (English-speaking IQR = 7, 11 vs. non-English speaking IQR = 7, 10) (Table 4). Though some subdomains were addressed slightly less often in non-English speaking visits (e.g., decision-making, ACP, depression, sleep difficulties, hospice collaboration), there were no clinically significant differences by subdomain (Supplemental Table S3).

### 3.5. Differences in Domains of Care by Patient/Clinician Race Concordance

There were 4538 (60%) visits with race discordance between the patient and documenting clinician, and 3010 (40%) visits that were race-concordant. Among visits coded as race-concordant, most were NHW clinicians with NHW patients (60%; Supplemental Table S4). One more subdomain was addressed in race-discordant encounters (9, IQR 7, 11) than in race-concordant visits (8, IQR 7, 10) (*p* < 0.001) (Table 5). While some differences in subdomains achieved statistical significance, none reached clinical significance. Goals of Care subdomains, such as use of technology (13% vs. 7.6%), and areas of potential conflict (12% vs. 7.5%) were addressed more frequently in race-discordant visits (*p*-values < 0.001) (Figure 1; Supplemental Table S5).

## 4. Discussion

This study examined differences in domains of care addressed in palliative care visits among children, adolescents, and young adults with cancer by social determinants of health. While statistically significant differences existed by race/ethnicity, SDI, primary language, and patient/clinician race concordance in the number of total, Goals of Care, Symptom Management, and Care Coordination subdomains, these were often small and likely not clinically significant. Clinically significant differences in subdomains are highlighted in Box 1.

Box 1 **Clinically significant differences in subdomains.**

Race/Ethnicity:

• Decision making was addressed in fewer visits with Hispanic/Latino patients than other races (27% vs. 37–42%; *p* < 0.001).
• Nausea/vomiting was addressed in fewer visits with NHB patients than other races (20% vs. 30–33%; *p* < 0.001).
• Anxiety was addressed in more visits with NHW patients compared to other races (38% vs. 16–22%; *p* < 0.001).
• Depression/sadness was addressed in more visits with NHW patients compared to other races (13% vs. 4.6–8.6%; *p* < 0.001).
• Collaboration with family services was completed more often in visits with Asian patients compared to other races (71% vs. 51–59%; *p* < 0.001).

Social Deprivation Index:

• Nausea/vomiting was addressed in fewer visits with high-disadvantage patients (21% vs. 32–32%; *p* < 0.001).

Language:

• There were no clinically significant differences in subdomains by language.

Race Concordance:

• There were no clinically significant differences in subdomains by race concordance.

Disparities exist in EOL outcomes by race and ethnicity [16]. Prior studies indicate that White patients are more likely than their Black counterparts to die naturally at home [12,13,14], and Hispanic patients are less likely to receive inpatient PPC [17,18] than non-Hispanic patients. While there were minor variations by race/ethnicity in addressing code status, ACP, goals of care, artificial nutrition/hydration, and use of technology, no differences met our threshold for clinical significance. However, statistically and clinically significant differences emerged in Symptom Management subdomains such as nausea/vomiting, anxiety, and depression, which were less often addressed in visits with NHB and Asian patients compared to NHW and Hispanic/Latino patients. Disparities in appropriately recognizing and responding to symptoms may contribute to varying quality of care by race and untreated pain or mental health concerns [40,41]. Further research should investigate why clinicians were less likely to address symptoms in PPC visits across different racial groups.

Differences in EOL treatment and outcomes are influenced by a complex interplay of variables, including decision-making processes, personal preferences, and health literacy, and are likely not driven by the content of PPC visits alone [11]. For instance, NHB patients/families are disproportionately affected by limited health literacy, which mediates differences in EOL preferences by race and may be a stronger predictor of preferences than race [42,43]. Differences in receipt of high-intensity medical care for children with cancer that appeared to be based on race disappeared after adjusting for prognostic factors [25].

Differences in decision-making roles may also contribute to disparities. Hispanic/Latino and NHB parents may prefer more active decision-making roles, yet oncologists have greater difficulty accurately predicting their decision-making preferences compared to NHW parents [26]. Our finding that decision-making was addressed less often in visits with Hispanic/Latino families may explain some of this variation.

SDI is deeply intertwined with race and ethnicity. In prior studies, patients with lower socioeconomic status (SES) were less likely to receive subspecialty PPC [17,20], and more likely to receive higher intensity care at EOL [14,19], exacerbating financial toxicity among low SES families [44,45]. In our data, there were not enough differences in SDI groups to account for differences in EOL care. Other factors, outside what is addressed in PPC visits, may explain variation in PPC by SDI, including insurance coverage, healthcare access, and workplace flexibility [45]. Improved and systematic screening for social deprivation [44] can minimize barriers to receiving appropriate care. However, some minor differences emerged in subdomains by SDI. The role of spirituality was more often discussed in high-disadvantage visits. Patients at lower socioeconomic status, or high SDI, are more likely to be more religious and spiritual [46], which may lead to greater discussion about the role of religion and spirituality in coping or healthcare decisions during PPC visits. Black patients are also more likely to utilize faith and spirituality to cope with their cancer [47,48], and in our sample, NHB patients were also more likely to have higher disadvantage.

ACP and code status were addressed more often in low-disadvantage visits. This difference may be mediated by health literacy. Low SES is consistently associated with lower health literacy [49], and low health literacy is linked with reduced ACP knowledge and participation [50], which may result in decreased interest and participation in such conversations. Additionally, discussions about housing, food, and transportation insecurities allow for less time to address complex topics like ACP and code status.

Language [21,27,51] and race concordance [52] between families and clinicians also affect the quality of PPC delivered. Hispanic/Latino patients face additional barriers when seeing non-Spanish-speaking clinicians, with barriers to care including discrimination, inconsistent interpreter use, confusion about prognosis, and inadequate EOL anticipatory guidance [22,53]. Our entire PPC team was primarily English-speaking. Fortunately, our data indicate no clinical differences by language in the number of PPC domains addressed. However, our institution has broad access to in-person and virtual interpreter services, and differences may be exacerbated when these services are not available [51]. Consistent use of interpreters improves communication quality [51] and patient outcomes, with one study demonstrating fewer adverse events and intensive care unit transfers among children who received consistent visits with interpreters while hospitalized [54]. High-quality interpreter utilization is critical to mitigating disparities by language.

Existing research on race concordance is mixed, with few studies demonstrating consistent differences in communication quality by patient–clinician race concordance [55,56]. However, some differences have emerged, with race concordant visits rated higher in patient satisfaction, information-giving, partnership building, and participatory decision-making [56]. In our exploratory analysis of race concordance, we initially hypothesized that race-discordant visits would address fewer subdomains than race-concordant visits, as clinicians may be more uncomfortable broaching certain topics like code status or ACP, and patients may be less willing to share their concerns. We instead found that race-discordant visits on average addressed one more subdomain than concordant visits, with no clinically significant differences in subdomain distribution. This may result from generally poorer communication [56], greater disagreements between the family and medical team, or more time needed to arrive at a decision. Concordant visits may also confer greater baseline understanding or surmising of the family’s primary goals and concerns, thus addressing fewer subdomains each visit. Further research can better elucidate the relationship between race concordance, subdomains addressed, and communication quality.

### 4.1. Strengths and Limitations

This study has multiple important strengths and limitations. This study is strengthened by its large sample size of over 7000 patient visits, allowing detection of nuanced differences between groups with sufficient power. The sample was diverse, with robust representation across multiple sociodemographic variables, most prominently NHW, NHB, and Hispanic/Latino patients. The sample is also widely representative of most pediatric cancer patients in the state of Georgia, with a range of diagnoses and clinical presentations. The palliative care team consisted of a broad sample of physicians, nurse practitioners, and residents/fellows who participated in patient care and generated notes. Moreover, this study fills a needed gap in current research regarding differences in what is discussed during PPC visits.

Given our large sample size, many differences were identified as statistically significant but did not result in clinically meaningful differences. We attempted to mitigate this effect with a clinical significance threshold of 10% for subdomains. While this study examines multiple sociodemographic variables independently, these variables (race, SDI, and language) are deeply interconnected, as noted in our sub-analysis of race and SDI (Supplemental Table S1). Although there is value in understanding the individual contributions of each variable, differences must be interpreted within the broader context of these relationships. Variables like health literacy, insurance coverage, and healthcare access were not examined in this study, but likely mediate relationships between social determinants of health and PPC domains addressed, meriting further exploration. Institutional differences like access to interpreter services and patient and clinician demographics may limit generalizability. Notably, most Hispanic/Latino patients in this sample identified as White, limiting our ability to examine differences by race among those identifying as Hispanic/Latino. Patient diagnosis may also influence our results, as children with CNS and solid tumors are more likely to receive palliative care early, and children with leukemia/lymphoma often receive palliative care later in their disease course, altering the topics of discussion during visits [57]. Our study focused on the content of PPC visits, as judged by the documenting clinician rather than the patient. Clinicians may copy forward domains, limiting accuracy, although all documenting clinicians are trained on its use. Moreover, this study only examined the clinician’s record of what was discussed and did not explore patient-reported perspectives on the subjects addressed, or what they hoped would be addressed during a given visit. Future studies can incorporate patient-recorded outcome measures to ground study results in the patient experience and amplify patient voices. Domains were not linked to EOL outcomes; further research can examine the relationship between PPC visit content and care outcomes like satisfaction with care, decisional regret, quality of life, and EOL outcomes, including healthcare utilization.

We are also limited by the demographics of the patient sample, which includes few American Indian, Alaska Native, and Pacific Islanders, and the PPC team, which was predominantly NHW, a group that is overrepresented compared to our patient population. There were no Hispanic/Latino clinicians, limiting analyses by ethnicity and language. Future research can investigate whether PPC visit content varies among a more diverse clinical team.

### 4.2. Practical Implications

This study has multiple key implications for clinical practice. In terms of correcting existing disparities in PPC and EOL care by sociodemographic factors, it directs our attention away from the individual content of visits and instead towards broader differences, including but not limited to clinician biases and communication style, institutional policies, healthcare accessibility, and health literacy. Advocacy to improve healthcare access and early integration of PPC may narrow disparities over time. PPC clinicians can also intentionally attend to some of the differences noted in our study. For instance, clinicians may more proactively address symptoms like nausea/vomiting, anxiety, and depression with NHB patients and families, given differences in symptom management by race. Additionally, clinicians should always adhere to the use of certified interpreters when available to enhance conversations with their patients and mitigate disparities. In race discordant visits, clinicians can set visit agendas based on the patient’s primary concern and ensure those concerns are addressed before broaching further subdomains.

## 5. Conclusions

Despite PPC’s value in improving goals of care discussions, symptom management, and EOL care, disparities persist in access to PPC services and EOL outcomes related to social determinants of health. When comparing the domains of care addressed in palliative care visits with children, adolescents, and young adults with cancer across different race/ethnicity, SDI, language, and race concordance groups, some small differences emerged in frequency and subdomains addressed. Despite statistical differences, most differences were not clinically significant. One more subdomain was addressed in NHW visits compared to other races/ethnicities, likely driven by symptoms (i.e., nausea/vomiting, anxiety, depression) that were more often addressed in NHW visits. One more subdomain was addressed in the intermediate disadvantage group, with a few other differences by SDI. One more subdomain was addressed in race-discordant than concordant encounters. There were a few differences by SDI or language alone; however, both variables are closely related to race/ethnicity. While differences in domains addressed during PPC visits may contribute to disparities in PPC and EOL care, these likely emerge from a far more complex interplay of variables, including health literacy, patient and family biases and preferences, clinician biases and communication style, and systemic factors. Further research can qualitatively investigate how the content of PPC visits influences future outcomes like symptom management, healthcare utilization, and EOL care, with the ultimate goal of enhancing goal-concordant care for children with cancer and their families.

## Figures and Tables

**Table 1:** Patient demographics and clinical characteristics.

	N = 467	
	N	%
**Age at diagnosis, years (Median, IQR)**	10.4	(4.3, 15.1)
**Sex**		
Female	223	47.80%
Male	244	52.20%
**Race**		
White	270	57.80%
Black	166	35.50%
Asian ^a^	26	5.60%
American Indian | Alaska Native | Pacific Islander ^a^	3	0.60%
Unknown ^a^	2	0.40%
**Ethnicity**		
Non-Hispanic/Latino	384	82.20%
Hispanic/Latino	83	17.80%
**Patient reported language**		
English	425	91.00%
Spanish	35	7.50%
Other	7	1.50%
**Diagnosis type**		
CNS tumor	166	35.50%
Solid tumor	170	36.40%
Leukemia/lymphoma	131	28.10%
**Social Deprivation Index (SDI)** ^b^		
Tertile 1 (low disadvantage)	166	35.55%
Tertile 2	145	31.05%
Tertile 3 (high disadvantage)	156	33.40%
**Rural–Urban Commuting Area (RUCA) codes**		
Rural/Suburban	48	10.28%
Urban	419	89.72%
**Vital status**		
Alive	198	42.40%
Deceased	269	57.60%
**Total number of PPC visits per patient**, Median (IQR)	10	(4, 21)
Total number of inpatient visits, Median (IQR)	5	(1, 17)
Total number of outpatient visits, Median (IQR)	3	(0, 6)

Abbreviations: IQR, interquartile range; CNS, central nervous system; PPC, pediatric palliative care. ^a^ In the manuscript, Asian/American Indian/Alaska Native/Pacific Islander/Unknown were analyzed as a cohort due to the small numbers of visits in the latter groups. ^b^ Patients were split into SDI tertiles post SDI linkage.

**Table 2:** Differences in PPC domains and subdomains addressed by race/ethnicity.

Domain		Race/Ethnicity				
	Overall	Non-Hispanic White (NHW)	Non-Hispanic Black (NHB)	Hispanic/ Latino, Any Race	Asian, American Indian, PI, Unknown, Other, Non-Hispanic	*p*-Value ^2^
	N = 7548	N = 3238 ^1^	N = 2439 ^1^	N = 1445 ^1^	N = 426 ^1^	
**Goals of Care**	4 (3, 5)	4 (3, 5)	4 (3, 5)	4 (3, 5)	4 (3, 5)	**<0.001**
Accommodating to disease	6392 (85%)	2816 (87%)	2026 (83%)	1166 (81%)	384 (90%)	**<0.001**
Medical symptoms	6326 (84%)	2796 (86%)	1994 (82%)	1179 (82%)	357 (84%)	**<0.001**
Understanding of disease/situation	6335 (84%)	2692 (83%)	2101 (86%)	1163 (80%)	379 (89%)	**<0.001**
Goals of Care	4893 (65%)	2074 (64%)	1639 (67%)	894 (62%)	286 (67%)	**0.004**
Decision-making	2707 (36%)	1211 (37%)	935 (38%)	384 (27%)	177 (42%)	<0.001 *
Role of spirituality	661 (8.8%)	252 (7.8%)	268 (11%)	114 (7.9%)	27 (6.3%)	**<0.001**
Advance Care Planning	861 (11%)	373 (12%)	322 (13%)	125 (8.7%)	41 (9.6%)	**<0.001**
Code status	390 (5.2%)	147 (4.5%)	136 (5.6%)	81 (5.6%)	26 (6.1%)	0.2
Use of artificial nutrition/hydration	348 (4.6%)	153 (4.7%)	106 (4.3%)	38 (2.6%)	51 (12%)	**<0.001**
Areas of potential conflict	781 (10%)	428 (13%)	182 (7.5%)	135 (9.3%)	36 (8.5%)	**<0.001**
Use of technology	807 (11%)	333 (10%)	289 (12%)	159 (11%)	26 (6.1%)	**0.004**
Complementary and alternative medicine	17 (0.2%)	8 (0.2%)	9 (0.4%)	0 (0%)	0 (0%)	**0.08**
**Symptom Management**	3 (2, 4)	3 (2, 5)	2 (2, 4)	3 (2, 4)	2 (2, 3)	**<0.001**
Wellbeing	5947 (79%)	2629 (81%)	1891 (78%)	1061 (73%)	366 (86%)	**<0.001**
Pain	6234 (83%)	2695 (83%)	2015 (83%)	1204 (83%)	320 (75%)	**<0.001**
Nausea/vomiting	2134 (28%)	1047 (32%)	489 (20%)	472 (33%)	126 (30%)	<0.001 *
Anxiety	2010 (27%)	1240 (38%)	379 (16%)	311 (22%)	80 (19%)	<0.001 *
Fatigue/tiredness	1056 (14%)	532 (16%)	265 (11%)	211 (15%)	48 (11%)	**<0.001**
Appetite	726 (9.6%)	395 (12%)	185 (7.6%)	108 (7.5%)	38 (8.9%)	**<0.001**
Depression/sadness	677 (9.0%)	418 (13%)	113 (4.6%)	124 (8.6%)	22 (5.2%)	<0.001 *
Sleep difficulties	852 (11%)	471 (15%)	221 (9.1%)	129 (8.9%)	31 (7.3%)	**<0.001**
Constipation	976 (13%)	431 (13%)	291 (12%)	210 (15%)	44 (10%)	**0.038**
Difficulty eating	607 (8.0%)	325 (10%)	145 (5.9%)	94 (6.5%)	43 (10%)	**<0.001**
Dyspnea	799 (11%)	364 (11%)	250 (10%)	141 (9.8%)	44 (10%)	0.4
Weakness and mobility issues	128 (1.7%)	46 (1.4%)	55 (2.3%)	19 (1.3%)	8 (1.9%)	**0.06**
Agitation	616 (8.2%)	286 (8.8%)	178 (7.3%)	133 (9.2%)	19 (4.5%)	**0.003**
Diarrhea	186 (2.5%)	99 (3.1%)	28 (1.1%)	51 (3.5%)	8 (1.9%)	**<0.001**
**Care Coordination**	2 (1, 2)	2 (1, 2)	2 (1, 2)	2 (1, 2)	2 (1, 2)	**<0.001**
Communication with specialty services	7363 (98%)	3173 (98%)	2379 (98%)	1403 (97%)	408 (96%)	**0.023**
Collaboration with family services	4297 (57%)	1829 (56%)	1428 (59%)	737 (51%)	303 (71%)	<0.001 *
Hospice collaboration	936 (12%)	339 (10%)	379 (16%)	157 (11%)	61 (14%)	**<0.001**
Psychosocial assessment	368 (4.9%)	207 (6.4%)	91 (3.7%)	48 (3.3%)	22 (5.2%)	**<0.001**
School issues	96 (1.3%)	52 (1.6%)	30 (1.2%)	10 (0.7%)	4 (0.9%)	0.067
Community supports	210 (2.8%)	106 (3.3%)	80 (3.3%)	22 (1.5%)	2 (0.5%)	**<0.001**
Home care services	117 (1.6%)	55 (1.7%)	47 (1.9%)	10 (0.7%)	5 (1.2%)	**0.018**
Relationship with PCP	25 (0.3%)	13 (0.4%)	0 (0%)	3 (0.2%)	9 (2.1%)	**<0.001**
**Total domains**	8 (7, 11)	9 (7, 11)	8 (7, 10)	8 (7, 10)	8 (7, 11)	**<0.001**

Bolded *p*-values denote statistical significance, and * denotes clinical significance (≥10% difference between groups). Abbreviations: PI, Pacific Islander; PCP, primary care physician; PPC, pediatric palliative care. ^1^ n (%); Median (Q1, Q3). ^2^ Pearson’s chi-squared test, Fisher’s exact test, and Kruskal–Wallis rank sum test.

**Table 3:** Differences in PPC domains addressed by social deprivation index tertile.

Characteristic (Median, IQR)	Overall	Tertile 1 (Low Disadvantage)	Tertile 2	Tertile 3 (High Disadvantage)	*p*-Value ^1^
	N = 7548	N = 2318	N = 2476	N = 2754	
Total Domains	8 (7, 11)	8 (7, 11)	9 (7, 11)	8 (7, 11)	0.092
Goals of Care	4 (3, 5)	4 (3, 5)	4 (3, 5)	4 (3, 5)	0.049
Symptom Management	3 (2, 4)	3 (2, 4)	3 (2, 4)	3 (2, 4)	<0.001
Care Coordination	2 (1, 2)	2 (1, 2)	2 (1, 2)	2 (1, 2)	<0.001

^1^ One-way analysis of means.

**Table 4:** Differences in PPC domains addressed by patient’s primary language.

Characteristic (Median, IQR)	Overall	English Speaking	Non-English Speaking	*p*-Value ^1^
	N = 7499	N = 6700	N = 799	
Total Domains	8 (7, 11)	8 (7, 11)	8 (7, 10)	0.001
Goals of Care	4 (3, 5)	4 (3, 5)	4 (3, 5)	0.052
Symptom Management	3 (2, 4)	3 (2, 4)	3 (2, 4)	0.01
Care Coordination	2 (1, 2)	2 (1, 2)	2 (1, 2)	0.003

^1^ One-way analysis of means. Primary language was not available for 49 visits.

**Table 5:** Differences in PPC domains addressed by patient–clinician race concordance.

Characteristic (Median, IQR)	Overall	Discordant	Concordant	*p*-Value ^1^
	N = 7548 ^1^	N = 4538 ^1^	N = 3010 ^1^	
**Total Domains**	8 (7, 11)	9 (7, 11)	8 (7, 11)	<0.001
Goals of Care	4 (3, 5)	4 (3, 5)	4 (3, 5)	<0.001
Symptom Management	3 (2, 4)	3 (2, 4)	3 (2, 4)	<0.001
Care Coordination	2 (1, 2)	2 (1, 2)	2 (1, 2)	<0.001

^1^ Welch’s two-sample *t*-test.

## Data Availability

The data presented in this study are available in the Supplementary Material.

## Supplementary Materials

The following supporting information can be downloaded at: https://www.mdpi.com/article/10.3390/children12121694/s1, Supplemental Table S1: Social deprivation index scores by patient race/ethnicity. Supplemental Table S2: PPC subdomains addressed by social deprivation index tertile. Supplemental Table S3: PPC subdomains addressed by patient primary language. Supplemental Table S4. Patient–provider race concordance by patient race/ethnicity. Supplemental Table S5. PPC subdomains addressed by patient–provider race concordance.

## Abbreviations

The following abbreviations are used in this manuscript:

ACP	Advance Care Planning
CNS	Central nervous system
EMR	Electronic Medical Record
EOL	End of life
IQR	Interquartile range
LCSW	Licensed clinical social worker
NHB	Non-Hispanic Black
NHW	Non-Hispanic White
NP	Nurse practitioner
PPC	Pediatric palliative care
RUCA	Rural–Urban Commuting Area
SDI	Social deprivation index
SES	Socioeconomic status
ZCTA	Zip code tabulation area
