# Supplementary material for: Relationship Between Social Determinants of Health and Domains of Care Addressed During Pediatric Palliative Care Visits for Children with Cancer
Source: Children (Basel). 2025 Dec 16;12(12):1694. doi: 10.3390/children12121694 (PMC12731678; doi:10.3390/children12121694)
Supplement: Supplementary file 1 [file children-12-01694-s001.zip › children-3993737-supplementary.pdf]

Supplemental Table S1. Social Deprivation Index Scores by Patient Race/Ethnicity

**SDI Score**	**Overall**	**Non-Hispanic Asian/ Other**	**Non-Hispanic Black**	**Hispanic/ Latino**	**Non-Hispanic White**	**p-value**[2]
	N = 467	N = 27	N = 160	N = 83	N = 197	
95% CI	(49, 54)	(28, 50)	(58, 66)	(47, 60)	(41, 48)	<0.001
Mean (SD)	51.78 (28.16)	38.89 (27.67)	62.23 (26.42)	53.33 (29.34)	44.41 (26.28)	
Min, Max	2.00, 100.00	6.00, 99.00	9.00, 100.00	3.00, 100.00	2.00, 99.00	

[1] Abbreviations: CI = Confidence Interval
[2] One-way analysis of means

Supplemental Table S2. PPC Subdomains Addressed by Social Deprivation Index Tertile

Characteristic		SDI Tertile			p-value[2]
	Overall	Tertile 1 (Low Disadvantage)	Tertile 2	Tertile 3 (High Disadvantage)	
	N = 7,548[1]	N = 2,318[1]	N = 2,476[1]	N = 2,754[1]	
**Goals of Care**	4 (3, 5)	4 (3, 5)	4 (3, 5)	4 (3, 5)	**0.005**
Accommodating to disease	6,392 (85%)	1,983 (86%)	2,087 (84%)	2,322 (84%)	0.4
Medical symptoms	6,326 (84%)	1,920 (83%)	2,106 (85%)	2,300 (84%)	0.1
Understanding of disease/situation	6,335 (84%)	1,949 (84%)	2,092 (84%)	2,294 (83%)	0.5
Goals of care	4,893 (65%)	1,500 (65%)	1,511 (61%)	1,882 (68%)	**<0.001**
Decision making	2,707 (36%)	897 (39%)	770 (31%)	1,040 (38%)	**<0.001**
Role of spirituality	661 (8.8%)	181 (7.8%)	172 (6.9%)	308 (11%)	**<0.001**
Advance Care Planning	861 (11%)	307 (13%)	282 (11%)	272 (9.9%)	**<0.001**
Code status	390 (5.2%)	143 (6.2%)	108 (4.4%)	139 (5.0%)	**0.017**
Use of artificial nutrition/hydration	348 (4.6%)	105 (4.5%)	142 (5.7%)	101 (3.7%)	**0.002**
Areas of potential conflict	781 (10%)	178 (7.7%)	296 (12%)	307 (11%)	**<0.001**
Use of technology	807 (11%)	224 (9.7%)	266 (11%)	317 (12%)	0.11
Complementary and Alternative Medicine	17 (0.2%)	3 (0.1%)	10 (0.4%)	4 (0.1%)	0.073
**Symptom Management**	3 (2, 4)	3 (2, 4)	3 (2, 4)	3 (2, 4)	**<0.001**
Wellbeing	5,947 (79%)	1,892 (82%)	1,953 (79%)	2,102 (76%)	**<0.001**
Pain	6,234 (83%)	1,867 (81%)	2,063 (83%)	2,304 (84%)	**0.007**
Nausea/vomiting	2,134 (28%)	743 (32%)	801 (32%)	590 (21%)	**<0.001***
Anxiety	2,010 (27%)	550 (24%)	696 (28%)	764 (28%)	**<0.001**
Fatigue/tiredness	1,056 (14%)	322 (14%)	380 (15%)	354 (13%)	**0.034**
Appetite	726 (9.6%)	243 (10%)	278 (11%)	205 (7.4%)	**<0.001**
Depression/Sadness	677 (9.0%)	188 (8.1%)	204 (8.2%)	285 (10%)	**0.006**
Sleep difficulties	852 (11%)	318 (14%)	285 (12%)	249 (9.0%)	**<0.001**
Constipation	976 (13%)	261 (11%)	394 (16%)	321 (12%)	**<0.001**
Difficulty eating	607 (8.0%)	167 (7.2%)	262 (11%)	178 (6.5%)	**<0.001**
Dyspnea	799 (11%)	254 (11%)	260 (11%)	285 (10%)	0.8
Weakness and Mobility Issues	128 (1.7%)	36 (1.6%)	42 (1.7%)	50 (1.8%)	0.8
Agitation	616 (8.2%)	141 (6.1%)	247 (10.0%)	228 (8.3%)	**<0.001**
Diarrhea	186 (2.5%)	32 (1.4%)	123 (5.0%)	31 (1.1%)	**<0.001**
**Care Coordination**	2 (1, 2)	2 (1, 2)	2 (1, 2)	2 (1, 2)	**<0.001**
Communication with specialty services	7,363 (98%)	2,262 (98%)	2,416 (98%)	2,685 (97%)	>0.9

Collaboration with family services	4,297 (57%)	1,336 (58%)	1,335 (54%)	1,626 (59%)	**<0.001**
Hospice collaboration	936 (12%)	391 (17%)	216 (8.7%)	329 (12%)	**<0.001**
Psychosocial assessment	368 (4.9%)	111 (4.8%)	119 (4.8%)	138 (5.0%)	>0.9
School issues	96 (1.3%)	44 (1.9%)	16 (0.6%)	36 (1.3%)	**<0.001**
Community supports	210 (2.8%)	69 (3.0%)	71 (2.9%)	70 (2.5%)	0.6
Home care services	117 (1.6%)	41 (1.8%)	36 (1.5%)	40 (1.5%)	0.6
Relationship with PCP	25 (0.3%)	8 (0.3%)	16 (0.6%)	1 (<0.1%)	**<0.001**
**Total Domains**	8 (7, 11)	8 (7, 11)	9 (7, 11)	8 (7, 11)	0.15

Bolded p-values denote statistical significance, *denotes clinical significance (>= 10% difference between groups)

Abbreviations: PCP, primary care physician; PPC, pediatric palliative care

[1] n (%); Median (Q1, Q3)

[2] Pearson’s Chi-squared test; Fisher’s exact test; Kruskal-Wallis rank sum test

Supplemental Table S3. PPC Subdomains Addressed by Patient Primary Language

Characteristic				p-value[2]
	Overall	English	Non-English	
	N = 7,499[1]	N = 6,700[1]	N = 799[1]	
**Goals of Care**	4 (3, 5)	4 (3, 5)	4 (3, 5)	0.11
Accommodating to disease	6,383 (85%)	5,706 (85%)	677 (85%)	0.7
Medical symptoms	6,277 (84%)	5,595 (84%)	682 (85%)	0.2
Understanding of disease/situation	6,307 (84%)	5,624 (84%)	683 (85%)	0.3
Goals of care	4,864 (65%)	4,366 (65%)	498 (62%)	0.11
Decision making	2,704 (36%)	2,462 (37%)	242 (30%)	**<0.001**
Role of spirituality	661 (8.8%)	587 (8.8%)	74 (9.3%)	0.6
Advance Care Planning	858 (11%)	794 (12%)	64 (8.0%)	**0.001**
Code status	387 (5.2%)	349 (5.2%)	38 (4.8%)	0.6
Use of artificial nutrition/hydration	348 (4.6%)	325 (4.9%)	23 (2.9%)	**0.012**
Areas of potential conflict	776 (10%)	701 (10%)	75 (9.4%)	0.3
Use of technology	803 (11%)	716 (11%)	87 (11%)	0.9
Complementary and Alternative Medicine	17 (0.2%)	17 (0.3%)	0 (0%)	0.2
**Symptom Management**	3 (2, 4)	3 (2, 4)	3 (2, 4)	**0.008**
Wellbeing	5,899 (79%)	5,284 (79%)	615 (77%)	0.2
Pain	6,185 (82%)	5,526 (82%)	659 (82%)	>0.9
Nausea/vomiting	2,115 (28%)	1,908 (28%)	207 (26%)	0.13
Anxiety	1,963 (26%)	1,782 (27%)	181 (23%)	**0.017**
Fatigue/tiredness	1,056 (14%)	960 (14%)	96 (12%)	0.076
Appetite	726 (9.7%)	663 (9.9%)	63 (7.9%)	0.069
Depression/Sadness	677 (9.0%)	630 (9.4%)	47 (5.9%)	**0.001**
Sleep difficulties	837 (11%)	801 (12%)	36 (4.5%)	**<0.001**
Constipation	961 (13%)	824 (12%)	137 (17%)	**<0.001**
Difficulty eating	607 (8.1%)	543 (8.1%)	64 (8.0%)	>0.9
Dyspnea	799 (11%)	705 (11%)	94 (12%)	0.3
Weakness and Mobility Issues	128 (1.7%)	122 (1.8%)	6 (0.8%)	**0.027**
Agitation	616 (8.2%)	567 (8.5%)	49 (6.1%)	**0.023**
Diarrhea	186 (2.5%)	148 (2.2%)	38 (4.8%)	**<0.001**
**Care Coordination**	2 (1, 2)	2 (1, 2)	2 (1, 2)	**0.023**
Communication with specialty services	7,314 (98%)	6,537 (98%)	777 (97%)	0.6

Collaboration with family services	4,260 (57%)	3,810 (57%)	450 (56%)	0.8
Hospice collaboration	906 (12%)	845 (13%)	61 (7.6%)	**<0.001**
Psychosocial assessment	368 (4.9%)	338 (5.0%)	30 (3.8%)	0.11
School issues	96 (1.3%)	87 (1.3%)	9 (1.1%)	0.7
Community supports	210 (2.8%)	194 (2.9%)	16 (2.0%)	0.15
Home care services	117 (1.6%)	108 (1.6%)	9 (1.1%)	0.3
Relationship with PCP	25 (0.3%)	22 (0.3%)	3 (0.4%)	0.7
**Total Domains**	8 (7, 11)	8 (7, 11)	8 (7, 10)	**0.038**

Bolded p-values denote statistical significance, *denotes clinical significance (>= 10% difference between groups)

Abbreviations: PCP, primary care physician; PPC, pediatric palliative care

[1] n (%); Median (Q1, Q3)

[2] Pearson's Chi-squared test; Fisher's exact test; Kruskal-Wallis rank sum test

Supplemental Table S4. Patient-Provider Race Concordance by Patient Race/Ethnicity

**Characteristic**	**Overall**<br><br>N = 7,548[1]	**Patient-Provider Race Concordance**		**p-value**[2]
		Discordant<br>N = 4,538[1]	Concordant<br>N = 3,010[1]	
**Patient race and ethnicity**				<0.001
Asian/American Indian / PI/ Unknown/ Other, Non-Hispanic	426 (5.6%)	409 (9.0%)	17 (0.6%)	
Black, Non-Hispanic	2,439 (32%)	2,010 (44%)	429 (14%)	
Hispanic/Latino	1,445 (19%)	698 (15%)	747 (25%)	
White, Non-Hispanic	3,238 (43%)	1,421 (31%)	1,817 (60%)	

Abbreviations: PI, Pacific Islander

[1] n (%) of visits

[2] Pearson's Chi-squared test

Supplemental Table S5. PPC Subdomains Addressed by Patient-Provider Race Concordance

Characteristic		Patient-Provider Race Concordance		p-value[2]
	Overall	Discordant	Concordant	
	N = 7,548[1]	N = 4,538[1]	N = 3,010[1]	
**Goals of care**	4 (3, 5)	4 (3, 5)	4 (3, 5)	**<0.001**
Accommodating to disease	6,392 (85%)	3,844 (85%)	2,548 (85%)	>0.9
Medical symptoms	6,326 (84%)	3,844 (85%)	2,482 (82%)	**0.009**
Understanding of disease/situation	6,335 (84%)	3,785 (83%)	2,550 (85%)	0.13
Goals of care	4,893 (65%)	2,946 (65%)	1,947 (65%)	0.8
Decision making	2,707 (36%)	1,611 (36%)	1,096 (36%)	0.4
Role of spirituality	661 (8.8%)	427 (9.4%)	234 (7.8%)	**0.014**
Advance Care Planning	861 (11%)	595 (13%)	266 (8.8%)	**<0.001**
Code status	390 (5.2%)	274 (6.0%)	116 (3.9%)	**<0.001**
Use of artificial nutrition/hydration	348 (4.6%)	251 (5.5%)	97 (3.2%)	**<0.001**
Areas of potential conflict	781 (10%)	554 (12%)	227 (7.5%)	**<0.001**
Use of technology	807 (11%)	577 (13%)	230 (7.6%)	**<0.001**
Complementary and Alternative Medicine	17 (0.2%)	9 (0.2%)	8 (0.3%)	0.5
**Symptom Management**	3 (2, 4)	3 (2, 4)	3 (2, 4)	**<0.001**
Wellbeing	5,947 (79%)	3,690 (81%)	2,257 (75%)	**<0.001**
Pain	6,234 (83%)	3,800 (84%)	2,434 (81%)	**0.001**
Nausea/vomiting	2,134 (28%)	1,227 (27%)	907 (30%)	**0.003**
Anxiety	2,010 (27%)	1,083 (24%)	927 (31%)	**<0.001**
Fatigue/tiredness	1,056 (14%)	632 (14%)	424 (14%)	0.8
Appetite	726 (9.6%)	466 (10%)	260 (8.6%)	**0.019**
Depression/Sadness	677 (9.0%)	373 (8.2%)	304 (10%)	**0.005**
Sleep difficulties	852 (11%)	535 (12%)	317 (11%)	0.091
Constipation	976 (13%)	648 (14%)	328 (11%)	**<0.001**
Difficulty eating	607 (8.0%)	438 (9.7%)	169 (5.6%)	**<0.001**
Dyspnea	799 (11%)	567 (12%)	232 (7.7%)	**<0.001**
Weakness and Mobility Issues	128 (1.7%)	89 (2.0%)	39 (1.3%)	**0.028**
Agitation	616 (8.2%)	411 (9.1%)	205 (6.8%)	**<0.001**
Diarrhea	186 (2.5%)	143 (3.2%)	43 (1.4%)	**<0.001**
**Care Coordination**	2 (1, 2)	2 (1, 2)	2 (1, 2)	**<0.001**
Communication with specialty services	7,363 (98%)	4,442 (98%)	2,921 (97%)	**0.021**
Collaboration with family services	4,297 (57%)	2,647 (58%)	1,650 (55%)	**0.003**
Hospice collaboration	936 (12%)	589 (13%)	347 (12%)	0.061

Psychosocial assessment	368 (4.9%)	207 (4.6%)	161 (5.3%)	0.12
School issues	96 (1.3%)	66 (1.5%)	30 (1.0%)	0.082
Community supports	210 (2.8%)	151 (3.3%)	59 (2.0%)	**<0.001**
Home care services	117 (1.6%)	80 (1.8%)	37 (1.2%)	0.066
Relationship with PCP	25 (0.3%)	15 (0.3%)	10 (0.3%)	>0.9
**Total Domains**	8 (7, 11)	9 (7, 11)	8 (7, 10)	**<0.001**

Bolded p-values denote statistical significance, *denotes clinical significance (>= 10% difference between groups)

Abbreviations: PCP, primary care physician; PPC, pediatric palliative care

[1] n (%); Median (Q1, Q3)

[2] Pearson's Chi-squared test; Fisher's exact test; Kruskal-Wallis rank sum test
